# Nutrigenomic evaluation of garlic (*Allium sativum*) and holy basil (*Ocimum sanctum*) leaf powder supplementation on growth performance and immune characteristics in broilers

**DOI:** 10.14202/vetworld.2017.121-129

**Published:** 2017-01-27

**Authors:** N. Sheoran, R. Kumar, A. Kumar, K. Batra, S. Sihag, S. Maan, N. S. Maan

**Affiliations:** 1 Department of Animal Nutrition, College of Veterinary Sciences, LLR University of Veterinary and Animal Sciences, Hisar - 125 004, Haryana, India; 2 Department of Animal Biotechnology, College of Veterinary Sciences, LLR University of Veterinary and Animal Sciences, Hisar - 125 004, Haryana, India

**Keywords:** broilers, garlic, gene expression, holy basil, toll-like receptors

## Abstract

**Aim:**

In this study, a planned research work was conducted to investigate the nutrigenomic aspects of supplementation of *Allium sativum* (garlic) and *Ocimum sanctum* (holy basil) leaf powder on the growth performance and immune characteristics of broilers.

**Materials and Methods:**

A 6 weeks feeding trial was conducted with 280-day-old Ven Cobb broilers, distributed randomly into seven experimental groups. Each treatment had 4 replicates with 10 birds each. The birds of the control group (T_1_) were fed a basal diet formulated as per BIS standards. The broilers of treatment groups T_2_ and T_3_ were fed basal diet supplemented with the commercially available garlic powder (GP) at levels of 0.5% and 1.0% of the feed, respectively, while broilers in T_4_ and T_5_ were fed basal diet supplemented with commercial grade holy basil leaf powder (HBLP) at levels 0.5% and 1.0% of the feed, respectively. Birds in the T_6_ were fed with 0.5% GP and 0.5% HBLP, whereas T_7_ was fed with 1.0% GP and 1.0% HBLP. At the end of the feeding trial (6^th^ week), blood samples were collected and analyzed for relative mRNA expression of toll-like receptors (TLR) 2, TLR 4 and TLR 7 using real-time polymerase chain reaction.

**Results:**

The mean body weight gain and feed conversion efficiency were improved (p<0.05) in broilers fed the GP and HBLP incorporated diets compared with the control group. The relative mRNA expression levels of TLR 2, TLR 4 and TLR 7 in the peripheral blood of the broilers were found to be increased (p<0.05) in the birds supplemented with graded levels of the GP and HBLP as compared to the untreated group.

**Conclusion:**

The present work concludes that the inclusion of GP and HBLP could enhance the production performance and immune status of birds by augmenting the T-cell mediated immune response and thereby protects them from disease without decreasing growth traits as a possible substitution to conventional antimicrobials.

## Introduction

Since time immemorial, traditional plants and their products, phytobiotics have been serving as an indispensable source of medicine in indigenous poultry production systems [1]. Today, around the globe, there is an increasing awareness about the emerging drug-resistant microbes, antimicrobial side effects and toxic residual effects of drugs in animal meat products which necessitate the search for newer feed alternatives or complementary medicines for the gut health maintenance and immunomodulation [2,3]. Therefore, the inherent utility of the indigenous herbal extracts/phytobiotics - such as *Allium sativum* (garlic), *Ocimum sanctum* (tulsi), *Curcuma longa*, *Azadirachta indica*, and *Withania somnifera* - are being searched out for the practical applications in poultry system for improving health and production indicating toward their immense therapeutic ability [4,5]. In the recent decade, there has been a vigorous emphasis on improving the growth and production performance in broiler industry which has adversely affected the immunological status of the broiler birds. This has thereby, altered the host defense mechanism by encountering the prevailing microbes such as bacteria, fungi, pathogenic viruses, endo and ecto-parasites, and several harmful toxins. Hence, researchers are now thinking toward the use of an array of antimicrobials of the herbal origin which have shown to possess multiple immunomodulatory actions such as phagocytosis, modulation of immunoglobulin and cytokine secretion, cellular co-receptor expression, class switching, lymphocyte expression, and histamine release [4].

Conventional herbal medicinal plants have been claimed to modulate the immune response, thereby augmenting non-specific immunity, essentially macrophages, granulocytes, natural killer cells and many complement functions [6,7]. Phytobiotics from herbs by acting as immunomodulators, serve as a potential alternative for the conventional chemotherapy in a variety of challenges by enhancing the natural defense mechanisms of the host. Several plant extracts, compounds, and formulations have also been patented including various polysaccharides, lectins, flavonoids, peptides, and tannins which are used in various *in* *vitro* models to assess their immune response [8]. The herbal preparations such as GP and HBLP have tremendous potential in improving the cell mediated immunity in poultry birds thereby benefiting the poultry sector immensely.

In the present experiment, we have investigated whether the *A. sativum* (garlic powder [GP]) and *O. sanctum* (holy basil leaf powder [HBLP]) incorporated diets, in compliance with maintaining growth and production performance could increase the relative mRNA expression of toll-like receptors (TLR 2, TLR 4 and TLR 7) thus enhance immunological status of the broilers by modulating their immune response.

## Materials and Methods

### Ethical approval

The animal experiment was conducted in accordance with guidelines approved by the Institutional Animal Ethics Committee, 12/CPCSEA Dated 8.4.2013 in the Department of Animal Nutrition, Lala Lajpat Rai University of Veterinary and Animal Sciences, Hisar.

### Birds, experiment design, and management

A total of 280-day-old commercial broiler chicks (Ven Cobb) maintained for a period of 6-week in the Department of Animal nutrition, were randomly allotted to 1 of 7 treatments in a completely randomized design. Each treatment consisted of 4 replicate pens with 10 chicks in each. The birds of the control group (T_1_) were fed a basal diet formulated as per BIS standards [9] meeting the requirements of the growing phase. The ingredient and chemical composition of the basal diet as analyzed according to the standards laid down by Association of Official Analytical Chemists(AOAC) [10] and is presented in Table-1. Seven dietary treatments included the basal diet (T_1_) which acted as control group. The basal diets supplemented with 0.5% and 1.0% of the commercially available garlic powder (GP) in treatment groups T_2_ and T_3_, respectively. Whereas, the birds in the treatment groups T_4_ and T_5_ were fed at 0.5% and 1.0% of the commercial grade HBLP, respectively. Birds in the treatment group T_6_ was fed with 0.5% GP and 0.5% HBLP, whereas T_7_ treatment group was fed with 1.0% GP and 1.0% HBLP. The experimental design is presented in Table-2. The basal diets were formulated to meet the requirements for growing phase of the broilers as per the standards recommended by the Bureau of Indian Standards (BIS) (Table-1) [9]. All the herbal preparations used in the present experiment were purchased from the local market. Floor litter system was followed where the chicks were kept hygienically in separate pens. All the birds were reared adopting uniform management conditions. The chicks were brooded at 35°C during the 1^st^ week and thereafter the temperature was reduced by 3°C every week until the temperature reached 25°C±1°C. The birds were vaccinated against prevailing diseases adopting a standard protocol. Individual body weight of chicks was recorded at 0 day age and thereafter weekly. Total feed consumed, growth rate, and feed conversion ratio (FCR) were calculated and presented in Table-2. At the end of the experiment, one bird from each replicate was slaughtered ethically by mechanical stunning followed by exsanguinities. Different carcass parameters - such as dressed weight (g), eviscerated weight (g), non-edible weight (g), and drawn weight (g) - were calculated and depicted in Table-3.

**Table-1:** Ingredient (%) and chemical composition (% dry matter [DM] basis) of basal diet.

Item	Starter diet	Finisher diet
Ingredient composition (%)		
Maize	53	57
Soybean meal	19	16
Rice police	3	4
Ground nut cake	12	11
Fish meal	7	5
Soybean oil	4	5
Mineral mixture	2.0	2.0
*Feed additives	0.29	0.29
Chemical composition (% DM basis)		
Crude protein	22.04	20.08
Crude fiber	3.61	3.32
Ether extract	8.38	8.98
Total ash	6.18	5.86
**Metabolizable energy (kcal/kg)	3056	3163

* Feed additives include Vitamin mixture-I - 10 g, vitamin, amino acid and Ca mixture-II - 20 g, coccidiostat (Dinitro-0-yoluamide) - 50 g, choline chloride - 50 g, Lysine - 50 g, DL- methionine - 80 g and chlortetracycline - 33.5 g/100 kg;

** Calculated values - BIS (2007)

**Table-2:** Experimental design.

Treatment groups	Particulars	Number of replicates	Number of birds/replicate	Total
T_1_	Control-standard broiler diet as per BIS (2007) specifications	4	10	40
T_2_	Control+5 g garlic powder/kg of diet	4	10	40
T_3_	Control+10 g garlic powder/kg of diet	4	10	40
T_4_	Control+5 g holy basil leaf powder/kg of diet	4	10	40
T_5_	Control+10 g holy basil leaf powder/kg of diet	4	10	40
T_6_	Control+5 g garlic powder/kg of diet+5 g holy basil powder/kg of diet	4	10	40
T_7_	Control+10 g garlic powder/kg of diet+10 g holy basil powder/kg of diet	4	10	40
			Total	280

**Table-3:** Performance parameters viz. feed intake (g/bird), body weight gain (g/bird) and FCR in broilers under different dietary treatments during overall period of 6 weeks of experiment.

Treatments	Feed intake (g/bird)	Body weight gain (g/bird)	FCR
T_1_	3771.75±4.23	1801.75^a^±1.79	2.09^c^±0.01
T_2_	3713.25±11.94	1823.00^ab^±21.17	2.03^bc^±0.02
T_3_	3687.25±79.93	1873.50^b^±9.13	1.96^b^±0.03
T_4_	3645.75±61.90	1818.50^ab^±11.92	2.00^bc^±0.03
T_5_	3634.25±17.80	1863.25^b^±8.41	1.95^b^±0.01
T_6_	3627.50±6.64	2129.25^c^±22.83	1.70^a^±0.01
T_7_	3640.25±4.23	2094.75^c^±32.93	1.73^a^±0.04

a,b,c Mean values bearing different superscripts in a column differ significantly (p<0.05); T_1_: Basal diet, T_2_: 0.5% garlic powder, T_3_: 1% garlic powder, T_4_: 0.5% tulsi leaf powder, T_5_: 1% tulsi leaf powder, T_6_: Mixture of garlic powder and tulsi leaf powder at 0.5% each, T_7_: Mixture of garlic powder and tulsi leaf powder at 1% each, FCR=Feed conversion ratio

### Blood collection and analysis

At the end of the feeding trial (6^th^ week), blood samples were collected from one broiler per replicate, making four samples per treatment and thus a total of 28 samples were analyzed. About 2 ml of blood was collected from each bird via brachial wing vein puncture using sterilized syringes and 5 ml scalp vein needle set into vacutainer containing ethylene diamine tetraacetic acid (EDTA) for TLR mRNA expression. Plasma was prepared by centrifuging the blood at 3000 rpm for 10 min. The plasma was then transferred into a microcentrifuge tube using a Pasteur pipette and stored at −20°C until further analysis.

### Reverse transcription (cDNA synthesis); RNA extraction and preparation of cDNA

Total RNA was isolated from blood samples by using TRIzol^®^ as per the manufacturer’s instruction. In brief, 1 ml of TRIzol^®^ reagent, 200 µl of chloroform was added to 600 ul of blood followed by centrifugation for phase separation and precipitation with isopropanol. Total RNA extracted was dissolved in 30 µL NFW and quantified using Qubit^®^ 2.0 fluorometer (Invitrogen). Reverse transcription was carried out with total reaction volume of 20 μL using cDNA synthesis kit (Fermentas). Briefly, NFW (7.00 μL), 5X RT buffer (4 μL), 10 mM dNTPs (Fermentas) (2 μL), total RNA (5 μL), RT 200 IU/μL (1 μL), Random hexamer (1 μL). The polymerase chain reaction (RT-PCR) cyclic conditions were as initial incubation at 25°C for 5 min, reverse transcription at 42°C for 1 h, and deactivation at 70°C for 5 min in thermal cycler (Applied Biosystem). The cDNA was stored at −20°C till further use.

### Real time PCR

For the analysis of temporal expression profile of different genes, real-time PCR was carried out using Step I plus real-time PCR system. For the real-time PCR reaction, SYBR Green dye based PCR mastermix (Affymetrix) was used, and all the instructions were followed as per the manufacturer. The reaction for the target gene, TLRs (TLR 2, TLR 4, and TLR 7), and the endogenous control, β-actin gene was carried out in triplicate along with non-template control as a negative control for each sample. The reaction mixture used to carry out the real-time PCR reaction for TLRs 2, 4 and 7; and β-actin gene contains 2X SYBR green PCR mastermix (Affymetrix, 12.5 μL), primers (forward and reverse 0.3 M each), NFW (variable), and template (2 μL). The cyclic conditions used for amplification were according to the instructions of the manufacturer. Amplification was done with denaturation for 15 min at 95°C, followed by 40 cycles of denaturation for 5 s at 95°C, and annealing/elongation for 30 s at 60°C, and a final melting curve analysis. The set of primers used for the real-time PCR is as shown in Table-4.

**Table-4:** Oligonucleotide sequences of sense and antisense primers for real-time PCR products determined.

Gene^1^	Primer	Primer sequence^2^	Accession No.	Product size
β-Actin	Sense	5′-GAGAAATTGTGCGTGACATCA-3′	L08165	152
	Antisense	5′-CCTGAACCTCTCATTGCCA-3′		
TLR 2	Sense	5′-CATTCACCATGAGGCAGGGATAG-3′	AB046533	157
	Antisense	5′-GGTGCAGATCAAGGACACTAGGA-3′		
TLR 4	Sense	5′-TTCAGAACGGACTCTTGAGTGG-3′	AY064697	131
	Antisense	5′-CAACCGAATAGTGGTGACGTTG-3′		
TLR 7	Sense	5′-TTGCTGCTGTTGTCTTGAGTGAG-3′	AJ627563	182
	Antisense	5′-AACAACAGTGCATTTGACGTCCT-3′		

1 TLR 2=Toll-like receptor 2; TLR 4=Toll-like receptor 4; TLR 7=Toll-like receptor 7.

2 Primers for toll-like receptors and β-actin were described by Sato *et al*. [27] and Bai *et al*. [28], respectively

### Relative quantification by comparative C_T_ method (ΔΔC_T_ method)

The average C_T_ (Threshold cycle) value obtained for the TLRs 2, 4 and 7 (target) gene was normalized to β-actin (endogenous control). The data obtained were subjected to comparative C_T_ method [11] for the analysis of the expression levels of targeted TLR gene and an endogenous control. The sample at 26 h of incubation was selected as calibrator.

### Sequencing of product

Amplicons were sequenced using the Big Dye Terminator Cycle Sequencing Kit (Applied Biosystems, Carlsbad, CA, USA) on an automatic ABI 3130 xl Genetic Analyzer (Applied Biosystems, Carlsbad, CA, USA). The sequence obtained shows 100% nucleotide identity with the TLR sequence of chicken available in the global database.

### Statistical analysis

Data were analyzed by one-way ANOVA as a completely randomized design using the General Linear Model (GLM) procedure of SAS Institute. Individual cage was used as an experimental unit for analyzing the performance data, whereas each bird selected was used as the statistical unit for analyzing the data of gene expression. Differences among means were tested by the least significant difference method, and p<0.05 was considered to be statistically significant group.

## Results

### Growth performance and FCR

Data pertaining to the growth performance as depicted in Table-3 revealed that significantly (p<0.05) highest mean body weight gain was observed in the treatment groups T_6_ and T_7_, where birds were supplemented a combination of GP and HBLP at 0.5 g and 1.0 g/kg of the diet, respectively. It was then followed by treatment groups T_3_ and T_5_ in which hens were fed ration supplemented with GP and HBLP at higher levels of inclusion of 1.0 g/kg of feed, respectively. While, the control group T_1_ recorded lowest mean body weight gain among all the treatments at all age groups. No significant difference in body weight gain was observed among the dietary treatments T_1_, T_2_, and T_4_. However, no significant (p<0.05) difference in feed intake (g/bird) was observed between dietary treatment groups T_2_, T_3_, T_4_, T_5_, T_6_ and T_7_ as compared to the control group T_1_ over the entire period of the experiment (Table-3).

The current research work on the FCR as shown in Table-3 unveiled that FCR was found to be significantly (p<0.05) improved in T_6_ and T_7_ as compared to control group T_1_ due to dietary supplementation of GP and HBLP in combination at 0.5% and 1.0% of the feed, respectively. Similarly, significantly improved FCR was observed in T_3_ and T_5_ treatment groups where GP and HBLP were included in the diet of hens at 1.0% of the ration, respectively. However, no significant difference was observed among supplemental groups T_2_ and T_4_ compared to T_1_ (control).

The study results as depicted in Table-5 revealed that overall with respect to the whole period of experiment there was significant (p<0.05) increase in the carcass parameters, viz., dressed weight (g), eviscerated weight (g) and drawn weight (g) in treatment groups T_3_, T_6_ and T_7_, in which broilers were supplemented with GP either alone or in combination with HBLP. While no significant difference was observed on the non-edible weight (g) due dietary supplementation of either of the herbal products (GP and HBLP).

**Table-5:** Carcass parameters viz. dressed weight (g), eviscerated weight (g), non-edible weight (g) and drawn weight (g) in broilers under different treatments.

Treatments	Dressed weight (g)	Eviscerated weight (g)	Non-edible weight (g)	Drawn weight (g)
T_1_	1284.85^a^±7.84	1071.59^a^±5.41	503.75±2.12	1108.07^a^±6.43
T_2_	1326.42^ab^±19.97	1093.38^ab^±21.25	452.97±1.59	1189.50^ab^±18.49
T_3_	1395.67^b^±8.39	1170.62^b^±7.99	519.62±2.79	1264.05^b^±10.39
T_4_	1301.25^ab^±13.65	1095.64^ab^±12.96	510.23±1.73	1187.84^ab^±9.49
T_5_	1366.23^b^±10.24	1159.87^ab^±9.37	484.89±0.94	1254.89^b^±14.83
T_6_	1613.48^c^±20.98	1367.78^c^±23.74	521.64±2.46	1478.33^c^±18.99
T_7_	1573.85^c^±35.24	1335.40^c^±31.48	516.21±1.94	1443.91^c^±25.31

a,b,c Mean values bearing different superscripts in a column differ significantly (p<0.05); T_1_: Basal diet, T_2_: 0.5% garlic powder, T_3_: 1% garlic powder, T_4_: 0.5% tulsi leaf powder, T_5_: 1% tulsi leaf powder, T_6_: Mixture of garlic powder and tulsi leaf powder at 0.5% each, T_7_: Mixture of garlic powder and tulsi leaf powder at 1% each

### TLR mRNA gene expression of broilers

**Figure-1:**
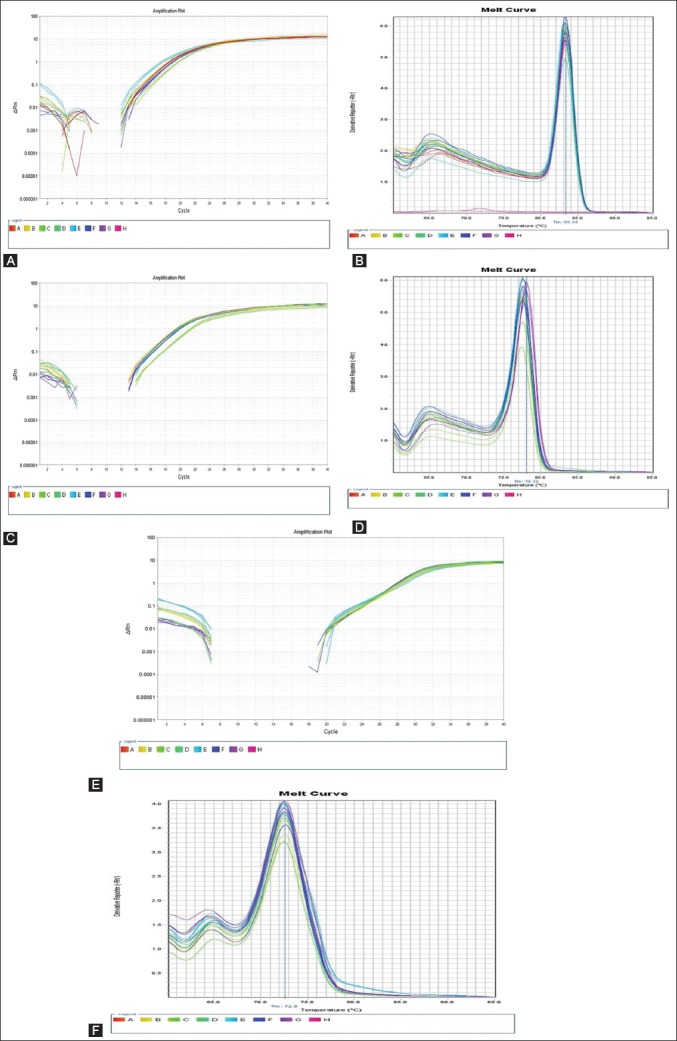
Amplification plot and melt curve for chicken toll-like receptors (TLR 2), TLR 4 and TLR 7. Panel A and B Amplification plot and melt curve for chicken TLRs 2. Panel C and D Amplification plot and melt curve for chicken TLRs 4. Panel E and F Amplification plot and melt curve for chicken TLRs 7.

**Figure-2:**
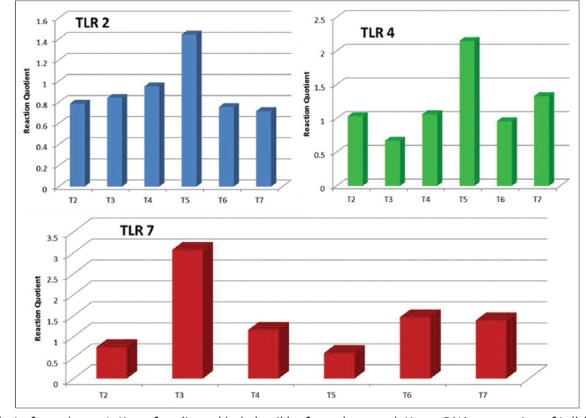
Effect of supplementation of garlic and holy basil leaf powder on relative mRNA expression of toll-like receptors 2 (TLR 2), TLR 4 and TLR 7 in the plasma of broilers. Significantly highest level of increase in the mRNA expression of TLR 2 and TLR 4 was observed in the treatment group T_5_. While, significantly highest increase in the mRNA expression of TLR 7 was observed in the treatment group T_3_ where broilers were fed garlic powder (GP) at 1% of the diet followed by the birds in the treatments groups T_6_ and T_7_ fed with increasing levels of 0.5% and 1% of the GP and holy basil leaf powder in combination, respectively.

The differential expression level of TLRs, viz. TLR 2, TLR 4 and TLR 7 gene transcripts in the of Ven Cobb commercial broiler strains was studied by relative quantification method. The level of target mRNA in different treatment groups was determined by comparative C_t_ method (ΔΔC_t_ method). The nutrigenomic expression analysis as presented in Table-6 and also depicted in Figures-1 and 2 revealed that relative mRNA expression of TLR 2 of broilers was found to be (p<0.05) enhanced in the treatment groups T_4_ and T_5_ supplemented with 0.5% and 1% of the HBLP fed individually or in combination with similar levels of garlic powder in treatment groups T_6_ and T_7_, respectively. While, as presented in Table-6 and shown in Figures-1 and 2 at the end of the 6 weeks of experimental protocol broilers had (p<0.05) higher relative mRNA expression of TLR 4 in the plasma of broilers fed diet supplemented with 0.5% and 1% of the HBLP in the treatment groups T_4_ and T_5_, respectively. Furthermore, the relative TLR 4 mRNA expression was found to be significantly higher in the treatment groups T_6_ and T_7_ where the birds were fed a combination of GP and HBLP at 0.5% and 1% in their diet, respectively. While no significant effect was observed in the relative mRNA expression of TLR 4 in the birds supplemented with 0.5% and 1% of the garlic powder in the treatment groups T_2_ and T_3_, respectively as compared to that of birds fed maize based basal diet in control T_1_ and other dietary treatments. However, the data pertaining to the relative mRNA levels of TLR 7 (Table-6) and shown in Figures-1 and 2 in the plasma of birds revealed slightly different pattern of expression and it was found that significantly highest levels of expression was observed in treatment group T_3_ supplemented at 1% of the garlic powder, followed by the treatment groups T_6_ and T_7_ in which the broilers were fed a combination of GP and HBLP at 0.5% and 1% of their ration, respectively. While no significant differences were observed in the experimental groups T_2_, T_4_ and T_5_ as compared to the control group and rest of the supplemental groups. However, treatment groups T_6_ and T_7_ did not show any significant (p<0.05) differences among themselves. In nutshell, experimental treatments containing GP and HBLP either alone or in their combination in the broiler’s diet have potent immune modulating activity by showing stimulatory effect on relative mRNA expression of TLR 2, TLR 4 and TLR 7 of the commercial broilers.

**Table-6:** Relative quantitation expression analysis of the toll-like receptors (TLR 2, TLR 4 and TLR 7) with reference to the endogenous reference gene B actin.

Sample name	Target Name	Cт	Cт Mean	Cт SD	ΔCт mean	RQ
T1	TLR 2	16.567	16.544^c^	0.0297	−2.801	1.000
T2		16.455	16.387^b^	0.0591	−2.457	0.787
T3		17.838	17.759^e^	0.0695	−2.552	0.841
T4		16.409	16.476^bc^	0.1109	−2.728	0.950
T5		15.560	15.408^a^	0.1409	−3.330	1.443
T6		17.029	16.958^d^	0.0723	−2.396	0.755
T7		17.125	17.097^cd^	0.0248	−2.320	0.716
T1	TLR 4	18.563	18.5541^e^	0.0201	−0.791	1.000
T2		17.995	18.009^b^	0.0261	−0.834	1.030
T3		20.110	20.104^f^	0.1091	−0.206	0.666
T4		18.336	18.329^d^	0.0199	−0.875	1.060
T5		16.827	16.846^a^	0.0811	−1.893	2.146
T6		18.587	18.630^e^	0.0420	−0.724	0.954
T7		18.201	18.217^c^	0.0631	−1.200	1.327
T1	TLR 7	28.603	28.811^cd^	0.1825	9.4658	1.000
T2		28.506	28.727^bcd^	0.2148	9.8828	0.749
T3		27.854	28.158^a^	0.2643	7.8472	3.070
T4		28.352	28.454^abcd^	0.0901	9.2500	1.161
T5		28.557	28.920^d^	0.3323	10.180	0.609
T6		28.373	28.274^ab^	0.0860	8.9186	1.461
T7		28.093	28.409^abc^	0.4489	8.9919	1.388

a,b,c,d,e,f Mean values bearing different superscripts in a column differ significantly (p<0.05). TLR=Toll-like receptors

## Discussion

Beneficial effects of bioactive plant substances in animal nutrition may include the stimulation of appetite and feed intake, the improvement of endogenous digestive enzyme secretion, activation of immune responses and antibacterial, antiviral and antioxidant actions [12]. Thus, all the nutrients are directed toward growth promotion resulting in enhanced growth performance. The findings of the current work reported a significant (p<0.05) positive effect on average body weight gain by the supplementation of graded levels of the GP and HBLP either alone or in their combinations in commercial broilers at 2, 4 and 6 weeks of age. The improvement in weight gain of the birds using garlic in their rations may probably be due to the fact that allicin (an antibiotic substance found in garlic), inhibits growth of intestinal bacteria such as *Staphylococcus aureus* and *Escherichia coli* and inhibit aflatoxins producing fungi [13,14]. Resultantly, when the load of these bacteria in the intestine is low, birds may absorb more nutrients, leading to the improvement in weight gain of the birds using rations supplemented with *A. sativum*.

The basil plant possessing antioxidant properties results in increase in the digestive enzymes and decrease in bacterial activities and thus leading to muscle weight gain in broiler chicks [15]. Even the improvement in live body weight in broilers may be due to antibacterial effects related to garlic derivative propylpropane thiosulfonate (PTSO) that led to modulation of normal intestinal microflora by competitive exclusion and antagonism and thus improved nutrients digestibility in growing broilers [13,16].

The present investigation revealed that broilers supplemented with GP and HBLP at various levels and in their combinations led to utilization of their feed more efficiently than the birds fed ration without addition. The antibacterial properties of these herbal supplements resulted in better absorption of the nutrients present in the gut, finally leading to improved FCR. It can thus be concluded that there was significant positive effect on the average body weight and subsequent enhanced FCR due to supplementation of the diet with herbal products, GP and HBLP either individually or in combinations in the commercial broiler strains.

Medicinal herbs have shown to possess multiple immunomodulatory actions like phagocytosis, modulation of immunoglobulin and cytokine secretion, cellular co-receptor expression, class switching, lymphocyte expression, and histamine release [4]. In current work, it was observed that dietary inclusion of GP and HBLP either alone or in combination significantly increased the relative mRNA expression of TLR cell markers, which confirmed that these herbal feed additives could stimulate the T cell immune system in the plasma of broiler birds.

In the present investigation, we found that there was a significant increase in the relative mRNA expression of TLR 2 and TLR 4 in the plasma of the broilers fed diet supplemented with graded levels of the HBLP either alone or in combination with garlic powder. TLR 2 recognizes a variety of microbial components. These include lipoproteins/lipopeptides from various pathogens, peptidoglycan and lipoteichoic acid from Gram-positive bacteria [17]. TLR 4 is the principal receptor for lipopolysaccharide, which is a major component of outer membrane of gram-negative bacteria [18]. Several studies have shown that the essential oils and biologically active compounds in fresh leaves of *O. sanctum* are effective against bacteria such as *E. coli, Shigella* spp. *Salmonella typhi*, and *Pseudomonas aeruginosa* [19].

The antimicrobial action of essential oils in *O. sanctum* (Linn.) is attributed to monoterpene components which are mostly phenolic in nature. They exert membrane damaging effects to microbial strains and stimulate leakage of cellular potassium which is responsible for a lethal action related to cytoplasmic membrane damage [20]. Immunostimulant potential of ‘Tulsi’ is helpful in the treatment of immunosuppression. It shows its immunomodulatory effect by increase in interferon-γ, interleukin-4, T-helper cells, NK cells [21] thus reducing total bacterial count, increasing neutrophil and lymphocyte count and enhancing phagocytic activity and phagocytic index. Oil from ‘Tulsi’ seed can mediate GABAergic pathways and by this it can modulate both humoral and cell-mediated immunity [22]. Antimicrobial effects of basil essential oil could also be owed to the higher concentrations of linalool and eugenol [23]. Another study revealed that the ethanol and methanol extracts of *O. sanctum* had the ability to inhibit the growth of all test bacteria including *E. coli* and *P. aeruginosa* [24]. Herbs can influence selectively the microorganism by an antimicrobial activity thus favors better nutrient utilization and absorption or the stimulation of the immune system [25].

From the above reported studies and our result findings, it can be inferred that, supplementation of diet with 1% HBLP and GP improved performance, as holy basil leaf might have suppressed the growth of harmful organisms like *Coliforms*, thereby creating a conducive environment for the growth of the beneficial microbes like *Lactobacillus, Bifidobacteria* spp. and thereby, aid in digestion and give better performance. Similar mechanism of action would have occurred while supplementing GP and HBLP in combination at 0.5% and 1% dose level. Result findings related to the relative mRNA gene expression of TLR 2 and TLR 4 in the present study it can be inferred that HBLP given at 1% of feed showed better results as compared to the GP fed at 1% of the feed.

TLR 7 family is implicated in intracellular recognition of nucleic acids. The TLR 7 recognizes some antiviral compounds and single-stranded viral RNA. In this study, supplementation of diet with garlic powder either individually or in combination with HBLP significantly increased the relative mRNA expression of TLR 7 in the plasma of the broiler birds. Researchers are focusing on an extract of *A. sativum* called Ajoene, which appears to protect CD+ cells from attack by HIV early in the viral life cycle. Allicin present in the *A. sativum* can protect against plasmodium infection by enhancing the host innate as well as innate immunity [26]. Tulsi or holy basil is suggested to shorten the course of illness, clinical symptoms in patients suffering from viral hepatitis and also enhances survival of viral encephalitis patients. Based on the above discussion and our present research work, it can be concluded that GP and HBLP both individually or in combination possess potent antiviral properties reflected by the increased expression of TLR 7 in the plasma of the broilers.

## Conclusions

Conventional medicinal herbs constitute an important aspect of applied biotechnological research and therefore as opposed to antimicrobial drugs and chemotherapeutic agents it can be employed for growth promotion and immunity booster in commercial broiler production systems. To summarize, the results of this study may lead us to conclude that the addition of GP and HBLP at higher level of 1% of the feed either alone or in combination in the diet of the broilers increased the relative mRNA expression of TLR 2, 4 and 7, although, among the two feed additives HBLP fed at 1% of diet was found to have better expression and production enhancing profile. Thus, the inclusion of the GP and HBLP could enhance the overall growth performance and immune status of birds by augmenting the T cell mediated immune response and thereby protects them from disease without decreasing performance traits.

## Authors’ Contributions

This study is the part of M.V.Sc. research work of the first author NS, who carried out the research under the guidance of Professor NSM. SM, AK and SS helped during the trial. The article was drafted by NS. The revision was made by KB, RK and SM. All authors have read and approved the final version of the manuscript.
